# Editorial: Selective and secretory autophagy pathways and molecules in the prevention and treatment of complex endocrine-metabolic diseases of aging

**DOI:** 10.3389/fendo.2023.1138630

**Published:** 2023-01-18

**Authors:** Maria Ines Vaccaro, Claudio Daniel Gonzalez, Vincenzo De Tata, Jorge Quarleri, Mauricio Fernando Budini

**Affiliations:** ^1^ National Scientific and Technical Research Council (CONICET), Institute of Biochemistry and Molecular Medicine Prof Alberto Boveris, University of Buenos Aires, Buenos Aires, Argentina; ^2^ Traslational Medicine Unit, Norberto Quirno Medical Education and Clinical Research Center (CEMIC), Buenos Aires, Argentina; ^3^ University of Pisa, Pisa, Italy; ^4^ National Scientific and Technical Research Council, Institute of Biomedical Research in Retroviruses and AIDS (INBIRS), University of Buenos Aires, Buenos Aires, Argentina; ^5^ Dentistry Faculty, Institute in Dentistry Sciences, Cellular and Molecular Biology Laboratory, University of Chile, Santiago, Chile

**Keywords:** oral diseases, thyroid cancer, vascular senescence, longevity, osteoarthritis, aging

Complex diseases are caused by a combination of genetic, environmental, and lifestyle factors. Many degenerative, metabolic, inflammatory diseases, cancers, and infections are included in this group of entities. Autophagy is known to be dysregulated in these pathological situations, some of them related to aging (Zhang et al.). Multiple different functions of the autophagy pathway or specific autophagy proteins are likely to be contributory to novel therapeutic, diagnostic, or preventative strategies (1). Autophagy is a cellular catabolic process that sequesters and delivers cytoplasmic components to the lysosome for degradation. There are three main forms of autophagy: (i) micro-autophagy refers to the direct sequestration of cytosolic components by the lysosome; (ii) the chaperon-mediated autophagy involves the translocation of specific proteins across the lysosome membrane through the receptor LAMP2A; and (iii) macroautophagy, where cargoes are sequestered within a unique double-membrane vesicle called the autophagosome, which fuses with the lysosome to deliver the inner vesicle in the degradative compartment (2).

By recycling cytoplasmic constituents, autophagy controls cellular bioenergetics and tissue remodeling. In addition, autophagy allows the selective elimination of misfolded proteins, protein aggregates, damaged organelles, intracellular pathogens, and lipid droplets. This pathway is called selective autophagy and has high relevance in the cell response to disease (1). Independently of the lysosomal degradation, the autophagic machinery can be involved in other non-degradative processes, such as the unconventional secretion by secretory autophagy, the mechanism of phagocytosis, and the regulation of inflammatory signaling (Gonzales et al.). As a result of the broad range of cellular functions, selective and secretory autophagy pathways, as well as autophagic proteins, play a key role in aging and have been linked to a wide range of cancers, infections, neurodegenerative disorders, metabolic diseases, inflammatory diseases, and muscle diseases (3).

Osteoarthritis is the most prevalent joint condition in older people and is essentially characterized by the increasing destruction of articular cartilage and subchondral bone, increased release of inflammatory factors, and reduced collagen formation. The preservation of normal expression and cartilage structure depends on chondrocytes. Because of their accelerated catabolic metabolism and suppressed anabolic metabolism, which leads to the secretion of more metal matrix proteins and the synthesis of fewer collagen and proteoglycans, they are the only cartilage cells that play a significant role in the pathogenesis of osteoarthritis. In osteoarthritis, chondrocytes produce more osteopontin (OPN), which stimulates the overexpression of matrix metalloproteinase and proinflammatory factors that trigger chondrocyte death. On the other hand, because autophagy safeguards both cellular function and survival, it is associated with osteoarthritis, cell death, and the slow deterioration of cartilage. Using an *in vitro* model, Bai et al. demonstrate that OPN can signal autophagy suppression in chondrocytes by interacting with the V3 integrin and CD44 cellular receptors and reducing the expression of the proteins BECN1 and LC3 II. The MAPK signaling pathway, particularly the ERK protein, is involved in such a mechanism. Furthermore, Bai et al. demonstrated that OPN also using the ERK MAPK pathway boosts chondrocyte over proliferation propitiating osteoarthritis. These results unveil a potential target of osteoarthritis therapy to counteract the cartilage-degrading processes using pharmacological therapies that activate autophagy.

Hamada et al. explore the role of a new system of mitochondrial quality control called “non-canonical mitophagy” in thyroid oncocytic tumors, whose cells are characterized by a large amount of abnormally enlarged mitochondria. In this system, a key role is played by the mitochondria-eating protein (MIEAP) which is thought to be a tumor suppressor and is not expressed in thyroid oncocytic tumors. It was therefore assumed that the loss of MIEAP expression could induce the accumulation of mitochondria in these tumors. By using genetically engineered mice, the authors showed that thyroid cancer development was accelerated only when the expression of both MIEAP and AGT5 (a component of autophagy machinery which is involved in canonical mitophagy) was suppressed, indicating that both molecules are probably tumor suppressors. However, tumors that develop following KO of both MIEAP and AGT 5 are of the non-oncocytic type. Hamara et al. conclude that, while having an important role in thyroid carcinogenesis, the impairment of mitophagy alone is not sufficient to induce the oncocytic phenotype.

Cardiovascular diseases (CVDs) cause around 17.9 million deaths (2019). Heart failure (HF) is the most prevalent CVD and is characterized by the inability of the heart to pump enough blood to the body, causing oxygen deficiency in different organs. HF can be classified according to the percentage (%) of ejection fraction (EF: blood pumped out from the heart) as i) reduced EF (<40%, HFrEF), ii) mid-range EF (40-50%, HFmrEF) and iii) preserved EF (>50%, HFpEF). Sanhueza-Olivares et al. reviewed the relationship between HFpEF, senescence, and autophagy. In addition to this, the authors make a very well description about how the micro and macrovascular components contribute to the HFpEF pathophysiology. The article mentions that senescence, considered a permanent arrest growth, has been identified in aged cells of the vascular system, including vascular smooth muscle cells (VSMC) and endothelial cells (EC). With this regard, senescence in VSMC and EC is related with loss in their physiological function and, probably, with the progression of HFpEF. On the other hand, autophagy, by recycling cellular proteins and organelles to maintain cell homeostasis, has been suggested to regulate senescence in VSMC and EC cells. Sanhueza-Olivares et al. highlight that a decrease in the autophagy activity is observed in VSMC and EC senescent cells and that the inhibition of autophagy activity by different mechanisms triggers senescence in VSMC and EC. Importantly, the revision article mentions that activation of autophagy can rescue the VSMC and EC from a senescent condition. Finally, through the analysis of adequate scientific literature, the review concludes that several HFpEF risk factors (diabetes, obesity, aging and hypertension) can impair autophagy and consequently contribute to the senescent status of the VSMC and EC cells in HFpEF. Sanhueza-Olivares et al. propose that the modification of autophagy activity, and consequently, of the senescent condition, can be considered as a target to protect against HFpEF.

Caries, periodontal diseases (including periodontitis), tooth loss, xerostomia, oral preneoplastic lesions, and oral cancer increase their frequency with age significantly affecting the quality of life and, in some cases, reducing life expectancy. Oral aging is associated with increased reactive oxygen species production as well as with reduced antioxidant defense. Oxidative stress and other degenerative mechanisms determine autophagy dysregulation which is closely linked with several age-related oral cavity diseases. Peña-Oyarzun et al. review the role and nature of these alterations in autophagy intensity and flux as well as describe their participation in the pathophysiology of periodontitis, periapical lesions, and oral cancer. They also outline the interaction between these diseases, dysfunctional autophagy, and the oral environment, including the loco-regional microbiome.

Defined as a multi-tasking adaptive cellular degradation and recycling strategy, the association between autophagy and longevity is reviewed in deep by Locatelli and Cenci. The role of autophagy in hyper-longevous mammals is a topic of special interest from different viewpoints. From physiology to pathophysiology; from prevention to treatment of age-related diseases. Departing from our taxonomic proximity to some hyper-longevous mammals (including bats) Locatelli and Cenci propose an interesting set of updated information opening the door to new insights as well as the detection of some scientific gaps to be filled towards a better understanding of human aging and age-related disorders.

This Topic aims to unravel mechanisms, allow autophagy to prevent or treat different complex diseases. For example, lifestyles and nutritional factors, such as exercise and caloric restriction, may exert their known health benefits through the autophagy pathway. In this special issue, we compiled a variety of approaches that exemplify the potential relevance in the management of autophagic response in osteoarthritis, thyroid carcinogenesis, vascular senescence, oral diseases, and the interesting relationship between autophagy and longevity.

Several currently available drugs have been shown to enhance autophagy, and this action may be repurposed for use in novel clinical indications. The development of new drugs, more selective inducers of autophagy or the designing of precise strategies against autophagic molecules are expected to maximize clinical benefits while minimizing toxicity. This Research Topic highlighted some current approaches on autophagy pathways and molecules that could potentially be targeted for the prevention or treatment of complex diseases.

## Author contributions

All authors listed have made a substantial, direct and intellectual contribution to the work, and approved it for publication.
